# Multi-center retrospective study of children with sickle cell disease admitted to pediatric intensive care units in the United States

**DOI:** 10.1038/s41598-023-32651-z

**Published:** 2023-04-25

**Authors:** Nicholas A. Ettinger, Danielle Guffey, Shaniqua J. Anum, Titilope Fasipe, Julie Katkin, Saleh Bhar, Gladstone Airewele, Arun Saini, Venée N. Tubman

**Affiliations:** 1 Division of Pediatric Critical Care, Department of Pediatrics, Texas Children’s Hospital/Baylor College of Medicine, 6651 Main Street, MC: E1420, Houston, TX 77030 USA; 2 Institute for Clinical and Translational Research, Baylor College of Medicine, Houston, TX USA; 3 Division of Pediatric Hematology-Oncology, Department of Pediatrics, Texas Children’s Hospital/Baylor College of Medicine, Houston, TX USA; 4 Division of Pulmonology, Department of Pediatrics, Texas Children’s Hospital/Baylor College of Medicine, Houston, TX USA

**Keywords:** Risk factors, Outcomes research, Paediatric research

## Abstract

Data on outcomes and interventions for children with sickle cell disease (SCD) admitted to a pediatric intensive care units (PICU) are unknown. We provide the first comprehensive multi-center report on PICU interventions associated with death, the need for invasive respiratory support or stroke among critically ill children with SCD. We collected retrospective multi-center cohort data from January 1, 2012 to December 31, 2019 utilizing the Virtual Pediatric Systems, LLC database. We identified 3388 unique children with SCD, accounting for a total of 5264 PICU admissions from 138 PICUs. The overall mortality rate for the PICU admissions cohort was 1.8% (95/5264 PICU admissions, 95/3388 [2.8%] of all unique patients), the rate of needing of needing Invasive Respiratory Support (IRS, a composite category of exposure) was 21.3% (872/4093 PICU admissions with complete data) and the overall rate of stroke (ischemic or hemorrhagic) was 12.5% (657/5264 PICU admissions). In multivariable analysis adjusting for admission age category, sex, race/ethnicity, PRISM-3 score at admission, exposure to IRS, quartile of unit volume of patients with SCD, and patient origin, admitted children who needed invasive respiratory support (IRS) had higher adjusted odds ratios for mortality (adjusted odds ratio [aOR], 19.72; 95% confidence interval [CI] 8.98–43.29; p < 0.001), although admitted children > 2 years old had decreased aOR for needing IRS (aOR 0.25–0.62; 95% CI 0.16–0.94; p < 0.001–0.025). By contrast, admitted children > 2 years old had a strikingly increased aOR for stroke (aOR 7.57–16.32; 95% CI 2.25–52.15; p < 0.001). These groups may represent PICU-specific subsets of patients with SCD who are at higher risk for more serious illness and should deserve early consideration for referral to a pediatric institution providing comprehensive care for patients with SCD.

## Introduction

Sickle cell disease (SCD) is one of the most common monogenetic disorders worldwide affecting 1 in 365 births among African Americans in the United States^1^, an estimated 230,000 births annually in Sub-Saharan Africa^2,3^ and more than 55,000 births annually in the Eastern Mediterranean and India^3^. SCD is an inherited, chronic hemolytic anemia, complicated by recurrent episodes of severe pain, inflammation, and microvascular occlusion. SCD presents a complex spectrum of severity that ranges from minor to life-threatening^4–7^. SCD vaso-occlusion can affect all organs^6^, including bone (acute pain crisis, necrosis, osteomyelitis), spleen (severe anemia from acute splenic sequestration and chronic hemolysis, functional immunocompromise from splenic infarcts), lungs^8^ (acute chest syndrome [ACS], pneumonia, pulmonary embolism, pulmonary hypertension^9^), brain (stroke^10,11^), heart (pulmonary hypertension, pulmonary embolism), kidney (acute kidney injury^12^, chronic nephropathy), and liver (sickle cell hepatopathy^13^).

Survival of pediatric patients with SCD has improved^5,14,15^ over the last 50 years with a contemporary reported survival to age 18 years of 93.9–98.4%^14^. Despite decreased mortality, SCD morbidity remains high and little is published on pediatric critical care outcomes for patients with SCD. To date, the largest publication related to critically ill children with SCD is a retrospective observational study examining outcomes for children with acute chest syndrome (ACS) and factors associated with the need for mechanical ventilation. Comorbidities of obesity, obstructive sleep apnea, and heart disease during episodes of ACS were associated with an increased risk for mechanical ventilation^16^.

Absent from prior studies is an examination of risks of PICU-associated procedures and therapies. To address this gap, we analyzed clinical characteristics and outcomes of pediatric patients with SCD admitted to a PICU from the multi-center Virtual Pediatric Systems, LLC (VPS) database. As these have been undescribed previously, we aimed to: (1) characterize the demographic profile of pediatric patients with SCD in the PICU; (2) characterize the common diagnoses and PICU-specific procedures and therapies children with SCD admitted to a PICU are exposed to and; (3) identify associations between clinical variables and mortality, the need for invasive respiratory support (IRS), or stroke. We hypothesized that there would be significant center variation in SCD mortality.

## Results

### Characteristics of admissions

We obtained data from the VPS database on 5264 admissions, representing 3388 unique patients with SCD from 138 separate PICUs (Table 1, see “Methods” section for more information on VPS database). The cohort of admitted children was 46.9% female and > 65% of the patients were older than 6 years of age. Our cohort was primarily Black (n = 4010, 76.2%). Prior to admission, > 50% of patients were transferred to the PICU from a regular floor or admitted straight from an ED (Table 1). The South US Census Region accounted for 54.9% of SCD PICU admissions (Table 1). A large number were also from the Midwest (n = 1184, 22.5%). The total number of PICU admissions with SCD in the VPS database remained relatively constant from 2012 to 2019, despite increases in total PICU admissions reported to VPS (Supplementary Fig. 1a). Similarly, the number of unique patients with SCD admitted to a PICU remained relatively flat from 2012 to 2019, despite increasing total numbers of PICU admissions in the VPS database (Supplementary Fig. 1b). Patients with SCD represented 0.5–0.7% of all admissions in the VPS database for those years. There was also great variation in the number of admissions per center submitting data (Supplementary Fig. 2). Nearly 75% of the centers comprise the smallest quartile of admissions whereas 5 centers comprise the largest quartile of admissions (Table 1, Supplementary Fig. 2).

**Table 1:** Demographics of admissions cohort and analysis of SCD PICU mortality (N = 5264 admissions)^*^

Characteristics	OVERALL (N = 5264)*		SURVIVED (N = 5169)		DIED (N = 95)		p value^§^		
	Median	(IQR)	Median	(IQR)	Median	(IQR)			
Weight (kg)	28.1	(17.9,46.9)	28.1	(17.9,46.7)	35	(20.0,53.0)	**0.05**		
Height (cm)^†^	131.5	(110.0,153.0)	131.5	(110.0,153.0)	151	(114.0,163.0)	**0.01**		
Median number of sickle cell admissions per PICU from 2012 to 2019	94	(43,178)	94	(43.0,178.0)	90	(47.0,168.0)	0.91		

	Overall (N = 5264)		Survived (N = 5169)		Died (N = 95)		p value		
	N	%	N	%	N	%			
Total admission numbers	5264	100	5169	98.2	95	1.8	–		
**Age**							0.20		
Infant (30 days–< 2 year)	481	9.1	472	9.1	9	9.5			
Child (2–< 6 year)	1125	21.4	1110	21.5	15	15.8			
Child (6–< 12 year)	1857	35.3	1828	35.4	29	30.5			
Adolescent (12–18 year)	1801	34.2	1759	34.0	42	44.2			
**Gender**							1.00		
Female	2470	46.9	2425	46.9	45	47.4			
Male	2794	53.1	2744	53.1	50	52.6			
**Race**^‡^							0.46		
Black	4010	76.2	3932	76.1	78	82.1			
Hispanic	249	4.7	246	4.8	3	3.2			
Other/mixed	292	5.5	290	15.6	2	2.1			
Unspecified/missing	713	13.5	701	13.6	12	12.6			
**Patient origin prior to PICU admission**							**0.01**		
Regular floor	1694	32.2	1658	32.1	36	37.9			
Emergency department (ED)	1555	29.5	1527	29.5	28	29.5			
Transfer from outside hospital (OSH)	696	13.2	678	13.1	18	18.9			
Operating room	616	11.7	612	11.8	4	4.2			
Home/skilled nursing/other	435	8.3	433	8.4	2	2.1			
Step-down unit	185	3.5	179	3.5	6	6.3			
Procedure suite	68	1.3	68	1.3	0	0			
Another ICU/neonatal ICU (NICU)	15	0.3	14	0.3	1	1.1			
**Geographic patient origin (US census region)**							0.50		
South	2889	54.9	2839	54.9	50	52.6			
Midwest	1184	22.5	1165	22.5	19	20.0			
West	637	12.1	626	12.1	11	11.6			
Northeast	434	8.2	422	8.2	12	12.6			
International	120	2.3	117	2.3	3	3.2			

Number of sickle cell admissions per PICU-by quartiles			Overall (N = 5264)		Survived (N = 5169)		Died (N = 95)		p value
Quartile	Number of admissions	Number of centers	N	(%)	N	(%)	N	(%)	0.712
Q1	0–43	103	1383	26.3	1361	26.3	22	23.2	
Q2	44–94	20	1328	25.2	1300	25.1	28	29.5	
Q3	95–178	10	1410	26.8	1389	26.9	23	24.2	
Q4	179–474	5	1141	21.7	1119	21.6	22	23.2	

	Median	(IQR)	(Minimum, maximum)	p-value					
**Percentage of mortality per center—by quartiles**				**p < 0.001**					
Q1	0	(0, 2.38)	(0, 11.11)						
Q2	1.85	(0, 2.99)	(0, 6.67)						
Q3	1.8	(0.70, 2.38)	(0, 3.13)						
Q4	1.14	(0.21, 4.17)	(0.21, 5.07)						
**Percentage of IRS per center—by quartiles**				**p < 0.001**					
Q1	13.33	(6.90, 21.43)	(0, 100)						
Q2	16.42	(11.7, 22.22)	(3.70, 35.44)						
Q3	20.31	(15.49, 29.70)	(11.29, 32.14)						
Q4	6.82	(2.53, 23.19)	(2.53, 23.96)						
**Percentage of stroke per center—by quartiles**				**p < 0.001**					
Q1	9.09	(2.70, 15)	(0, 100)						
Q2	10.23	(6.58, 14.81)	(1.69, 22.22)						
Q3	16.9	(10.94, 20.72)	(4.03, 22.43						
Q4	8.71	(7.17, 14.52)	(7.17, 21.20)						

Significant values are in [bold].

*Totals include data from 138 centers representing 5264 ICU admissions from 3388 unique patients. If data was incomplete in the database, those reduced totals are noted.

^**†**^For height, total N = 2747 due to missing data.

^‡^VPS does not adhere to the standard definitions of race or ethnicity as defined by NIH, rather uses an internal definition.

^§^Data analyzed with two-sample Wilcoxon rank-sum (Mann–Whitney) [medians], exact testing or Chi-square (categorical data) or Kruskal–Wallis (the percentage distributions for each quartile). These data do not account for correlations within a patient or a center.

The primary admission diagnoses reported in the VPS database for this cohort largely aligned with SCD pathophysiology, although important deviations were seen. Sepsis and viral infections accounted for over 60% of reported primary infectious VPS diagnoses (Table S3). Though specific infectious pathogens were not always reported, pneumococcal infections were less frequently reported than gram-negative septicemia and salmonella infections. ACS was the leading primary reported respiratory diagnosis (59.6%, Table S4). Primary cardiac diagnoses were reported in less than 5% of all admitted children among patients with SCD in the VPS database (Table S5).

### Associations with mortality

**Figure 1:**
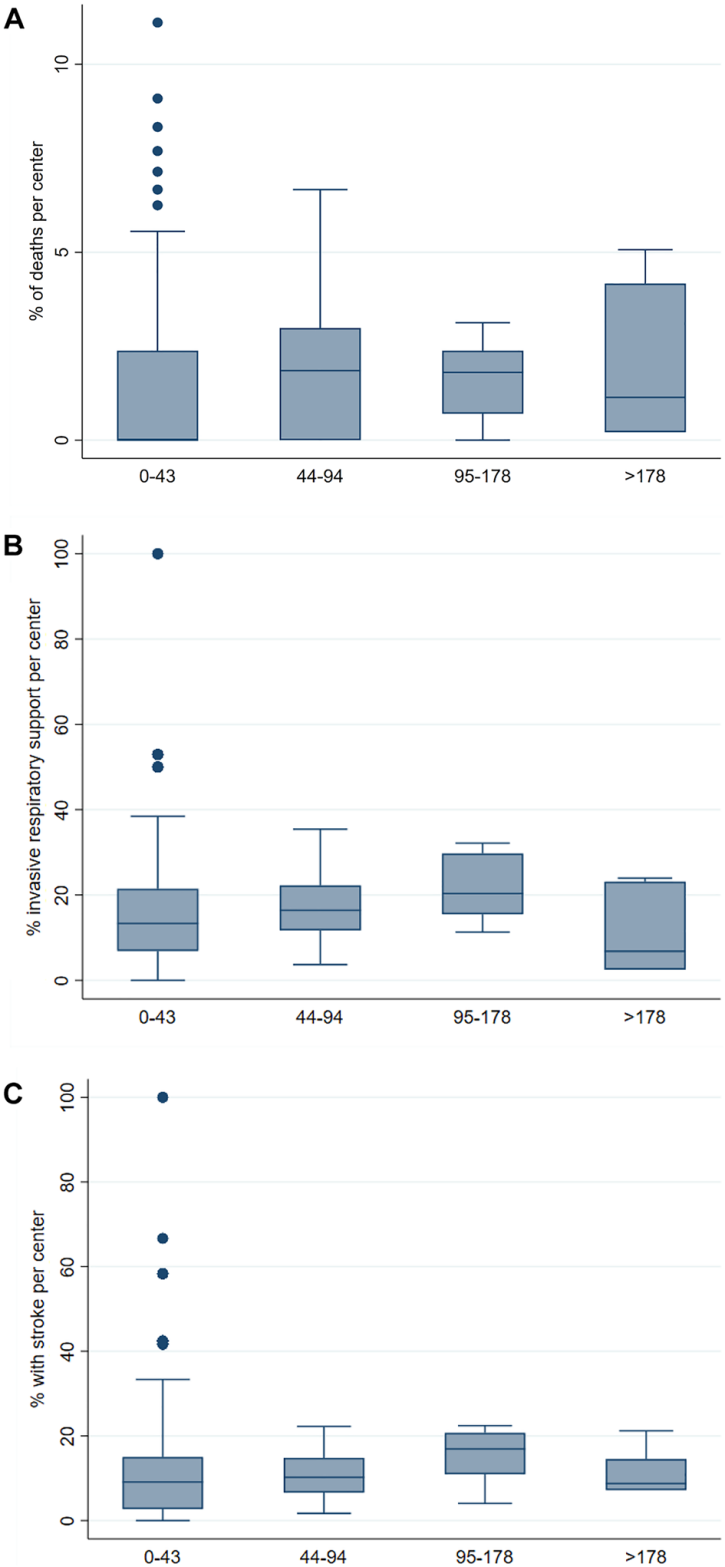
Box-whisker plot of percent of deaths per center, percent of patients with need for invasive respiratory support and percent of patients with stroke by quartiles of center volume. The vertical axis represents percentage of all patients reported from each center from 2012 to 2019. (**A**) Mortality; (**B**) need for invasive respiratory support or (**C**) stroke. The horizontal axis represents the number of admissions per center displayed by quartiles (see Table 1 for numeric quartile cutoffs and number of centers per quartile). The data are presented as box-whisker plots with the grey boxes representing the 25–75% percentiles of the data and the whiskers representing the 10% and 90% percentiles accordingly. Data outside the 90% are plotted individually. For each plot, by Kruskal–Wallis testing, the distributions for each quartile are different (p < 0.001).

We next compared the characteristics of patients with SCD who died versus those who survived PICU admission. Most patients with SCD admitted to the PICU survived their PICU admission. The overall mortality rate was 1.8% (1.8% for the entire cohort [95/5264 admissions], 2.8% [95/3388] of all unique patients; Table 1). Patients who died had higher median weight (n = 5264, 35 vs. 28.1 kg, p = 0.049) and height (n = 2747,151 vs. 131.4 cm, p = 0.009; Table 1). There were no differences in mortality by median number of SCD admissions per PICU, age category, gender, race/ethnicity, US Census Region, or quartile of SCD admissions per PICU (Table 1). The centers with the lowest overall volume had the widest distribution of mortality rates, with some smaller centers reporting mortality rates > 10% compared to less than ~ 4% for the larger centers (Table 1, Fig. 1a). A significantly higher proportion of admitted children who died in PICU were transferred from a regular hospital floor or from an outside hospital (p = 0.013; Table 1). Although the category of “Patient Origin prior to PICU Admission” appeared significant in initial analysis (Table 1), the univariate pairwise comparisons for location of origin did not identify a statistically significant ‘at risk’ location (Table 2).

**Table 2:** Univariate associations for mortality, need for invasive respiratory support (IRS) or stroke.

Variable		Mortality (N = 5264)		IRS (N = 4093)**		Stroke (N = 5264)**	
		Odds ratio (OR)*	p value (95% confidence interval [CI])	OR*	p value (95% CI)	OR*	p value (95% CI)
Weight		1.01	0.06 (1.00, 1.02)	**0.99**	**< 0.01 (0.98,1.00)**	**1.02**	**< 0.01 (1.00, 1.03)**
Height^†^		**1.01**	**0.03 (1.00, 1.02)**	**0.99**	**0.04 (0.99, 1.00)**	**1.00**	**0.08 (1.00, 1.01)**
**Age**			0.15		**< 0.01**		**< 0.01**
Infant (30 days–< 2 year)		*Reference*	–	*Reference*	–	*Reference*	–
Child (2–< 6 year)		0.71	0.43 (0.30,1.66)	**0.50**	**< 0.01 (0.34,0.75)**	**4.74**	**< 0.01 (1.68, 13.42)**
Child (6–< 12 year)		0.86	0.70 (0.40, 1.86)	**0.49**	**< 0.01 (0.34,0.72)**	**10.79**	**< 0.01 (3.86, 30.22)**
Adolescent (12–18 year)		1.33	0.45( 0.63, 1.42)	**0.36**	**< 0.01 (0.82,1.30)**	**11.70**	**< 0.01 (4.11, 33.34)**
Male		0.93	0.75 (0.61, 1.42)	1.04	0.77 (0.82,1.3)	0.90	0.70 (0.52, 1.55)
**Race**			0.51		**0.04**		0.86
Black		*Reference*	–	*Reference*	–	*Reference*	–
Hispanic		0.78	0.69 (0.23, 2.63)	0.77	0.46 (0.38, 1.55)	0.95	0.95 (0.20, 4.45)
Other/mixed		0.34	0.14 (0.08, 1.44)	0.59	0.08 (0.33, 1.06)	0.62	0.41 (0.20, 1.91)
Unspecified/missing		0.90	0.78 (0.45, 1.81)	**1.76**	**0.03 (1.05, 2.96)**	1.10	0.83 (0.47, 2.54)
PRISM-3		**1.21**	**< 0.001 (1.18, 123)**	**1.31**	**< 0.001 (1.26, 1355)**	**1.06**	**0.02 (1.01, 1.11)**
**Patient origin prior to PICU admission**			**0.05**		**< 0.01**		**< 0.01**
ED		*Reference*	–	*Reference*	–	*Reference*	–
Regular floor		1.18	0.51 (0.72, 1.95)	1.01	0.92 (0.78,1.33)	**0.57**	**< 0.01 (0.45, 0.74)**
Transfer from OSH		1.45	0.23(0.79, 2.64)	**1.80**	**< 0.01 (1.29, 2.51)**	1.04	0.82 (0.72, 1.52)
Operating room		0.36	0.06 (0.13, 1.02)	1.31	0.13 (0.92, 1.86)	1.25	0.10 (0.95, 1.64)
Home/skilled nursing/other		0.25	0.11 (0.05, 1.33)	0.45	0.09 (0.18, 1.13)	**0.77**	**0.01 (0.09, 0.77)**
Step down unit		1.83	0.18 (0.76, 4.41)	**1.76**	**0.05 (1.00, 3.10)**	0.75	0.28 (0.45, 1.26)
Procedure suite		1.00	< 0.01 (0,0)	1.18	0.70 (0.51, 2.77)	**3.42**	**< 0.01 (1.91, 6.13)**
Another ICU/NICU		3.90	0.20 (0.49, 30.67)	**8.65**	**0.01 (1.63, 45.87)**	0.91	0.90 (0.20, 4.05)
**Region**			0.83		0.26		0.67
International		*Reference*	–	*Reference*	–	*Reference*	–
South		0.75	0.68 (0.19, 2.92)	2.77	0.25 (0.48, 15.99)	0.39	0.38 (0.05, 3.22)
Midwest		0.63	0.52 (0.15, 2.59)	1.43	0.70 (0.24, 8.48)	0.25	0.21 (0.03, 2.20)
West		0.69	0.62 (0.16, 2.98)	1.33	0.75 (0.22, 8.03)	0.26	0.23 (0.03, 2.38)
Northeast		1.07	0.93 (0.24, 4.81)	1.03	0.97 (0.15, 7.17)	0.29	0.31 (0.03, 3.09)
**Number of sickle cell admissions per PICU- by quartiles**			0.73		**0.02**		**0.04**
Q1	0–43	0.68	0.38 (0.29, 1.62)	0.28	0.12 (0.06, 1.39)	0.99	0.97 (0.57,1.71)
Q2	44–94	0.93	0.86 (0.39, 2.20)	0.47	0.38 (0.09, 2.52)	0.97	0.93 (0.56, 1.68)
Q3	95–178	0.73	0.51 (0.29, 1.84)	1.20	0.84 (0.2, 7.00)	1.46	0.18 (0.84, 2.54)
Q4	> 178	*Reference*	–	*Reference*	–	*Reference*	–

Significant values are in [bold].

*Univariate odds ratios for mortality, need for IRS and stroke calculated with a mixed effect logistic regression model accounting for correlations within each patient (i.e., multiple admissions per patient) and accounting for correlations for within each institution (i.e., “center effect”).

**Invasive respiratory support (IRS) and stroke represent composite categories, see “Methods”.

^**†**^For height, total N_mortality_ = 2798, total N_IRS_ = 2507, total N_stroke_ = 2798 due to missing data.

The risk of death during admission was associated with specific primary reported diagnoses and with certain reported diagnosis categories (Table S6). Patients with SCD admitted to the PICU with a primary reported diagnosis in the cardiac disease, infectious disease, pulmonary hypertension, or hemorrhagic stroke categories had increased odds ratios for death (Table S6). The odds ratios for mortality during PICU admission for patients with SCD were highest for those with a primary or secondary cardiac diagnosis (OR 11.55, p < 0.001; Table S6). SCD admissions with a general diagnosis categorized as cardiac, infectious, respiratory, neurologic (including stroke), transplant, stroke (combination of ischemic and hemorrhagic), hemorrhagic stroke, ischemic stroke, pulmonary hypertension, or pulmonary embolism all had increased odds ratios for death in the PICU (Table S6). Interestingly, admitted children to the PICU with a primary sickle cell or respiratory diagnosis had lower risk for mortality suggesting either a protective effect or that these patients may have been less ill (OR 0.23, p < 0.001 and OR 0.43, p = 0.003; Table S6).

Certain procedures or therapies typically performed in the PICU were associated with mortality (Table S7) in our univariate analysis. Cardiac interventions such as a cardiopulmonary resuscitation (CPR), extracorporeal membrane oxygenation (ECMO) or an echocardiogram (OR 240.71, 30.15, 7.24 p < 0.001) or invasive respiratory interventions such as high frequency oscillation ventilation (HFOV, OR 24.71, p < 0.001)), inhaled nitric oxide (iNO, OR 7.89, p < 0.001), a chest tube (OR 4.66, p = 0.001), bronchoscopy (OR 3.89, p = 0.010), or tracheostomy (OR 3.49, p = 0.025) were associated with increased likelihood of death. More generally, the composite category of need for invasive respiratory support (IRS, OR 45.96, p < 0.001, see “Methods” for precise definition), the procedure of intubation specifically (OR 42.96, p < 0.001) and longer duration of mechanical ventilation (OR 1.03, p < 0.001) were associated with increased likelihood of death. In contrast, exposure to high-flow nasal cannula (HFNC) or non-invasive positive pressure ventilation (NIPPV) was not associated with higher likelihood of death (OR 0.86, 1.06, respectively; p > 0.5). Admissions that required any type of CT scan (OR 7.23, p < 0.001), any type of central line (OR 12.8, p < 0.001), an intraosseous catheter (OR 29.33, p < 0.001), or an arterial catheter (OR 14.07, p < 0.001) also had a higher likelihood of death.

Not all PICU interventions (procedures or therapies) were associated with increased odds ratio of death in patients with SCD. While exposure to any type of nasal feeding tube (nasogastric: OR 5.48, p = 0.007; nasoduodenal/nasojejunal: OR 18.05, p = 0.009) or a laparotomy (OR 3.78, p = 0.036) had higher odds ratios for death in the PICU, exposure to paracentesis, splenectomy, appendectomy, or bone marrow biopsy was not associated with increased mortality (Table S8). Exchange transfusion or plasmapheresis (the therapies, a composite category) did not confer additional risk, but patients requiring a central venous dialysis catheter (OR 2.31, p = 0.001) had a higher odds ratio for death. Similarly, admitted children requiring any variant of renal replacement therapy [composite category] (OR 23.73, p < 0.001) or those who specifically required continuous renal replacement therapy (CRRT, OR 42.47, p < 0.001) had elevated odds of PICU mortality. Those who underwent the procedure of placement of a peritoneal dialysis (PD) catheter or who required PD (the therapy) did not have increased risk of death. Electroencephalogram (EEG) monitoring (OR 3.4, p = 0.007), intracranial pressure monitoring (OR 7.85, p = 0.002), a pentobarbital coma (OR 42.34, p = 0.035), or hypothermic therapy (OR 98.63, p = 0.013) all had higher odds ratios for death; but exposure to a lumbar puncture, a shunt tap, spinal fluid diversion, cerebrovascular coiling, aneurysm repair, or a craniotomy did not confer additional risk.

In univariate analysis (Table S7), the odds of PICU mortality for admitted children with SCD increased with higher Pediatric Risk of Mortality-3 (PRISM-3)^17^ scores (OR 1.21, p < 0.001), increasing hospital length-of-stay (LOS, OR 1.01, p = 0.001), and increasing PICU LOS (OR 1.03, p < 0.001). In multivariable analysis (Table 3), as PRISM-3 scores increased by one unit, the adjusted odds ratio (aOR) for mortality increased by 1.13 and the aOR of mortality was 19.7 times higher for patients who required IRS compared to those who did not, after adjusting for age, sex, race/ethnicity, PRISM-3 score, IRS, unit volume of SCD admissions, and SCD patient origin.

**Table 3:** Multivariable model of odds ratio for mortality, need for invasive respiratory support (IRS) and stroke.

Variable		Mortality (N = 4093)		IRS (N = 4093)**		Stroke (N = 4093)**	
		Adjusted OR (aOR)*	p value (95% CI)	aOR*	p value (95% CI)	aOR*	p value (95% CI)
**Age**			0.53		** < 0.01**		**< 0.01**
Infant (30 days–< 2 year)		*Reference*	–	*Reference*	–	*Reference*	–
Child (2–< 6 year)		0.86	0.77 (0.31, 2.37)	**0.62**	**0.03 (0.40, 0.94)**	**7.57**	**< 0.01 (2.35, 24.33)**
Child (6–< 12 year)		1.23	0.65 (0.50, 3.04)	**0.58**	**0.01 (0.39,0.88)**	**16.64**	**< 0.01 (5.29, 52.36)**
Adolescent (12–18 year)		1.47	0.40 (0.60, 3.58)	**0.25**	** < 0.01 (0.16, 0.39)**	**16.32**	**< 0.01 (5.11, 52.15)**
Male		0.85	0.51 (0.52, 1.38)	0.99	0.92 (0.77, 1.27)	0.85	0.58 (0.48, 1.50)
**Race**			0.75		**0.01**		0.49
Black		*Reference*	–	*Reference*	–	*Reference*	–
Hispanic		1.13	0.87 (0.27,4.69)	0.72	0.39 (0.24, 1.52)	0.49	0.40 (0.10, 2.51)
Other/mixed		0.56	0.43 (0.13, 2.42)	**0.48**	**0.03 (0.26, 0.92)**	0.45	0.23 (0.12, 1.64)
Unspecified/missing		0.74	0.43 (0.35, 1.57)	**1.86**	**0.03 (1.08, 3.23)**	1.24	0.66 (0.48, 3.17)
PRISM-3 score		**1.13**	** < 0.01 (1.10, 1.15)**	**1.33**	** < 0.01 (1.28, 1.38)**	1.02	0.38 (0.97, 1.07)
IRS**		**19.72**	** < 0.01 (8.98, 42.39)**	N/A	N/A	**2.49**	**< 0.01 (1.39, 4.48)**
**Number of sickle cell admissions per PICU by quartiles**			0.39		**0.01**		0.08
Q1	0–43	0.67	0.30 (0.31, 1.42)	**0.18**	**0.05 (0.03, 0.96)**	0.31	0.11 (0.07, 1.30)
Q2	44–94	0.71	0.34 (0.35, 1.44)	0.29	0.17 (0.05, 1.72(	0.28	0.09 (0.06, 1.21)
Q3	95–178	0.53	0.08 (0.26, 1.09)	0.92	0.93 (0.14, 5.93)	0.87	0.86 (0.19, 4.02)
Q4	> 178	*Reference*	–	*Reference*	–	*Reference*	–
**Patient origin prior to PICU admission**			0.26		** < 0.01**		**< 0.01**
ED		*Reference*	–	*Reference*	–	*Reference*	–
Regular floor		1.37	0.34 (0.72, 2.60)	1.11	0.50 (0.82, 1.50)	**0.26**	**< 0.001 (0.15, 0.47)**
Operating room		0.45	0.17 (0.15, 1.42)	**1.87**	** < 0.01 (1.27, 2.77)**	0.62	0.19 (0.31, 1.27)
Other		1.21	0.58 (0.62, 2.35)	**1.60**	**0.01 (1.15, 2.24)**	0.57	0.07 (0.31, 1.06)

Significant values are in [bold].

*See “Methods” section for details of statistical analysis.

**Invasive respiratory support (IRS) and stroke represent composite categories, see “Methods”.

### Associations with IRS (composite category, see “Methods”)

Given that IRS was associated with increased mortality, we also examined risk factors associated with IRS (Table S9). Need for IRS was associated with smaller median weight and height, age category, race/ethnicity, patient origin prior to PICU admission, number of SCD admissions per PICU and PICU outcome. Need for IRS was not associated with gender or geographic origin (Table S9). In univariate analysis (Table 2), higher weight (OR 0.99, p = 0.001), height (OR 0.99, p = 0.035) and age categories > 2 years (OR 0.36–0.5, p < 0.001) all had ORs < 1, indicating a decreased need for IRS. The risk of needing IRS increased with higher PRISM-3 scores (1.31, p < 0.001) as well as with transfer from an outside hospital (OR 1.8 p = 0.001), a step-down unit (OR 1.76, p = 0.05), or another NICU or ICU (OR 8.65, p = 0.011). In our multivariable model (Table 3), not surprisingly, higher PRISM-3 scores conferred a greater risk of IRS (aOR 1.33, p < 0.001). Admissions with “Other/Mixed” race/ethnicity had adjusted OR < 1 (aOR 0.48, p = 0.03), whereas admissions with “Unspecified/Missing” race/ethnicity (aOR 1.86, p = 0.03), admissions from the OR (relative to the ED, aOR 1.87, p = 0.002) and admissions from “Other” (aOR 1.60, p = 0.006) had aORs > 1 for IRS. Children older than 2 years had aORs < 1 for IRS (aOR 0.25–0.62, p < 0.001 to p = 0.025), suggesting that those patients were at lower risk for IRS. Interestingly, admitted children from low-volume centers (relative to high-volume centers) had aORs < 1 for need for IRS (aOR 0.18, p = 0.045) although similar to mortality, the distributions of percentage of patients requiring IRS at smaller volume centers, compared to larger volume centers varied tremendously (Table 1, Fig. 1b).

### Associations with stroke (composite category, see “Methods”)

Lastly, we examined associations for stroke in our cohort as stroke causes major morbidity for pediatric patients with SCD (Table S10). Admitted children with stroke were heavier and taller and stroke admissions had higher proportions of children > 6 years old. Admitted children with stroke in the PICU had higher rates of mortality (3.8% vs. 1.5%, Table S10). In univariate analysis (Table 2), increased risk of stroke in patients with SCD in the PICU was associated with heavier patients (OR 1.02, p < 0.01), age category > 2 years (OR 4.74–11.70, p < 0.01), admission from a procedure suite (OR 3.42, p < 0.001), higher PRISM-3 Scores (OR 1.06, p < 0.02) or the overall category of median number of SCD admission per PICU (p < 0.042). By contrast, SCD admissions to the PICU from the regular floor (OR 0.57, p < 0.001) or from home/skilled nursing (OR 0.77, p = 0.01) had decreased risk for stroke. In our multivariable model (Table 3), relative to children < 2, older children had a much higher risk of stroke (aOR range 7.57–16.64, p < 0.001) along with children who required IRS (aOR 2.49, p < 0.01). Conversely, admitted children to the PICU that came from a regular hospital floor had a decreased aOR for the risk of stroke (aOR 0.26, p < 0.001). Interestingly, elevated PRISM-3 scores were not associated with higher risk of stroke in our multivariable model. Although the overall category of median number of admissions per center did not reach statistical significance for risk for stroke (Table 3), similar to both mortality and need for IRS, the percentage distributions by quartile demonstrated large amounts of variability comparing smaller volume centers to larger volume centers (Fig. 1c).

## Discussion

We examined PICU-associated risk factors and outcomes for pediatric SCD patients in a PICU using national, multi-center data. We found significant center variation in admissions and wide variation in mortality rates across center volume quartiles. Although overall cohort mortality was low, patients with SCD admitted to PICUs with increased admission acuity (higher PRISM scores), patients with need for major organ support (IRS, CRRT, ECMO) or who require significant PICU interventions (any type of nasal feeding tube, interventions required to treat significant neurologic injury) were all associated with significantly increased mortality risk.

We hypothesized that there would be significant center variability in outcomes for pediatric SCD patients admitted to the PICU. At the level of “number of admitted children per center” ,not surprisingly, our hypothesis was true^1^. Geographically, the Southern US Census region followed by the Midwest US Census region were the largest contributors to our data^1,18^. Centers in the smallest volume quartile had a wider distribution of mortality rates (as well as rates of needing IRS and rates of stroke, Fig. 1) with some centers experiencing PICU mortality rates > 10% whereas the overall cohort mortality rate was 1.8%. The distribution of pediatric oncology-related mortality in PICUs has also demonstrated regional differences in racial/ethnic mortality risks^19^. It is possible that smaller centers may be less well-resourced or less experienced caring for critically ill patients with SCD. Alternatively, at larger centers, the criteria for admission may be wider decreasing the in-hospital rates of mortality, IRS and stroke for those centers.

A complex picture emerged when we examined the relationship of patient origin prior to PICU admission to mortality, risk for IRS, and risk for stroke. Reassuringly, in both univariate analyses and our multivariable model, no specific patient origin had a higher likelihood of mortality. However, there were differences for need for IRS in both the univariate analysis and the multivariate model, with admission from the operating room or the composite category of “Other” having an increased aOR for need for IRS. The absence of a mortality signal related to pre-PICU patient origin may indicate that admission to the PICU is less likely to be based on local preferences than on disease-specific factors. In general PICU populations, using the same VPS database (compared to children admitted to the PICU from an ED), PICU admissions initially triaged to a regular floor have been shown to have higher mortality, longer PICU LOS, and higher rates of needing common PICU procedures^20^.

In our analysis, primary admission diagnoses that were cardiac- or pulmonary hypertension-related conferred increased odds ratios of death. A 2013 registry review describing outcomes for pediatric patients with SCD placed on ECMO reported 52% overall survival, with higher mortality for cardiac ECMO compared to respiratory ECMO^21^. These data are similar to other broader ECMO data in general pediatric populations. A registry review of 14,725 pediatric patients from the international Extracorporeal Life Support Organization database for 2009–2013 demonstrated that overall mortality was 43% and 49%, respectively, for pediatric respiratory and pediatric cardiac ECMO^22^.

Not surprisingly, ACS was the most frequently documented respiratory VPS diagnosis. An early study of 46 patients with SCD admitted to a PICU in the UK identified the most common reason for admission as ACS^23^. A retrospective observational study using the nationally representative Kids’ Inpatient Database found that comorbidities of obesity, obstructive sleep apnea, and heart disease during episodes of ACS were associated with increased risk for mechanical ventilation^16^. Our PICU-centric data shows that IRS and associated procedures did impart higher likelihood for PICU mortality. The risk for IRS was not distributed equally across age groups. The SCD PICU cohort was older with > 60% of admitted children over 6 years of age. As children became older and heavier, their likelihood of IRS decreased. These are challenging data to interpret because previous literature have characterized ACS to be more severe in older patients with SCD^24,25^. It is conceivable that for younger children with SCD admitted to the PICU with acute respiratory failure, the severity of illness may not be attributable as much to SCD, but rather to interactions between the inherent pathophysiology of their age and the primary cause of their acute respiratory failure (likely infectious). Previous prospective multinational studies have shown that critically ill children who are underweight have higher 28-day mortality than children who are normal weight or overweight/obese^26^, although other retrospective studies using primarily US data have not found any associations between body habitus and PICU mortality^27^.

Stroke, as a composite category of both ischemic and hemorrhagic stroke, was significantly associated with mortality (Table S6). When analyzed separately, hemorrhagic stroke had significantly higher risk for mortality compared to ischemic stroke in our data (Table S6). Previous work (see Fig. 2^28^) has shown a higher hazard rate of ischemic stroke in younger patients with SCD with that hazard rate waning in the teenage years accompanied by a concomitant rise in the hazard rate of hemorrhagic stroke. In our multivariable model for stroke (composite category), children > 2 years of age and children who required IRS had markedly elevated odds ratios for stroke. In pediatrics overall, the in-hospital mortality rate for acute ischemic stroke is 2.6%^29^ and in patients with SCD, primary hemorrhagic stroke has been associated with hypertension, recent exposure to corticosteroids and transfusions^30^. Previous work in general pediatric populations has shown that children in the Southeastern United States have higher stroke mortality rates compared to children from other US states and that children less than 4 years old had higher rates than older children^31^. Unfortunately, VPS does not capture important stroke prevention measures for patients with SCD (e.g., regular transcranial Doppler ultrasound, hydroxyurea therapy, chronic blood transfusions)^10^.

SCD admissions that required intensive central nervous system-oriented therapies all had extremely elevated likelihood of PICU mortality (Table S8). A common complication of severe neurologic injury is dysphagia and increased aspiration risk, perhaps explaining the relationship between nasal feeding tubes and PICU mortality in our cohort. In a retrospective study of almost 1000 children with neurologic impairment, 5 year survival after feeding tube placement was 75.8%^32^.

This study has several important limitations. Because VPS registry data is collected on a voluntary basis, internal funding challenges may prevent participating centers from submitting data every year. PICU admission criteria may vary between centers or by region. We attempted to account for this potential confounding by adjusting for “center effect” in our analysis. A few of the outcomes within our cohort were relatively rare, raising the small risk of over-stratification the data and the creation of spurious chance associations. In addition, it is possible that some patients with SCD were omitted from the data, or that relevant important diagnoses or co-morbidities or interventions for patients with SCD were either mis-recorded or not captured. However, the likelihood of this type of systemic error is low as data abstractors are trained, have critical care experience and there are internal quality-control processes for each contributing center. A potential misclassification that could affect analysis would be the mis-inclusion of sickle cell trait (who have much lower risks of organ failure) however we specifically excluded sickle cell trait from our VPS query and included all genotypes of SCD, which should lower that potential. For many patients, there was no height value included in the dataset preventing us from analyzing the effect of body mass index. Elevated PRISM-3 scores were associated with both mortality and IRS, but because PRISM-3 is designed as a PICU-centric illness severity score, this confounding is not surprising. Finally, neither sickle-cell specific treatments and monitoring nor preventive respiratory therapies nor simple blood product transfusions are captured in the VPS database.

In summary, our retrospective analysis of a large, multi-year, multi-center cohort of patients with SCD admitted to a PICU demonstrated that while overall mortality for PICU patients with SCD remains low, there is wide variety of outcomes across center size with more variability in mortality, need for IRS and risk of stroke in smaller volume centers compared to higher volume centers. In addition, there are specific categories of patients with SCD in the PICU (SCD patients in the PICU who are < 2 years of age, SCD patients who require intubation and mechanical ventilation, SCD patients with severe renal or neurologic failure) that should trigger concern for providers that a particular patient with SCD may be at higher risk for death or more serious illness. As not all pediatric SCD patients will present to a tertiary children’s hospital at first admission and analogous to recently published arguments that adult “patients with SCD have a substantial risk of adverse outcomes if they require ICU admission”^33^, our identified subsets of pediatric patients with SCD should deserve early consideration for referral to a pediatric institution providing full, comprehensive care for patients with SCD.

## Methods

### Design

We conducted a retrospective, multi-center observational cohort analysis of de-identified data from the VPS database. This study was approved by the Baylor College of Medicine Institutional Review Board (IRB), in accordance with the Helsinki Declaration (BCM IRB#:H-47768; Sickle Cell Disease in the Pediatric Intensive Care Unit: A Multi-center Analysis of Clinical Characteristics, Interventions and Outcomes; date: 6/9/2021). Due to the retrospective nature of the study, the requirement for informed consent was waived by the Baylor College of Medicine IRB. Virtual Pediatric Systems, LLC (VPS) is a multicenter PICU registry database that facilitates comparative pediatric critical care research, quality improvement efforts and benchmarking both within and among institutions^34^. More than 200 PICUs across North America voluntarily submit data and are included in the registry database. De-identified data are abstracted locally in real-time using a standardized data form with predetermined data elements by specially trained individuals with critical care experience and mapped using a standardized list of diagnoses after a quality-control process to minimize missing or incorrect data (https://myvps.org/#difference/; Tables S1–S5. The VPS dataset includes demographic information, geographic origin data^18^, PICU procedures and therapies performed, and severity of illness scoring data. Although there is a core dataset that all contributing centers must submit to participate in VPS, other data elements (e.g. height) are recommended but not required for all admissions.

### Outcomes

In the VPS database, demographic data such as age were potentially identifiable and thus were only provided as age categories. For each admission, a curated primary diagnosis and a list of secondary diagnoses for each admission are generated by the data abstractors (Tables S1–S5). Clinical data include de-identified VPS admission center label, primary diagnosis, secondary diagnoses, PICU mortality, PRISM-3 scores, PICU procedures and therapies performed, hospital length of stay (LOS), PICU LOS, and duration of mechanical ventilation. VPS diagnosis groupings are listed in Tables S1 and S2. The need for invasive respiratory support (IRS) was a composite category defined as exposure to the following VPS procedures: endotracheal intubation, invasive bilevel positive airway pressure (BPAP), invasive continuous positive airway pressure (CPAP), conventional mechanical ventilation (including CPAP plus Pressure Support), and high frequency oscillator ventilation (HFOV).

### Patients

We queried the VPS database for all patients admitted to PICUs from January 1, 2012 to December 31, 2019. PICU admitted children with either a primary or secondary diagnosis of SCD were included. We excluded patients < 1 month of age and > 18 years of age at the time of admission and any patient with a diagnosis of sickle cell trait or thalassemia.

### Statistical analysis

We used comparative statistics and characterized inter-institutional variability. To maximize the analytic power of our de-identified data, we analyzed our cohort at the level of admissions instead of unique patients. To generate odds ratios in univariate analysis, we used a mixed effect logistic regression model that accounted for correlations in the data for each patient (many patients had multiple admissions) and each center (i.e., “center effect”). We constructed a multivariable model incorporating age category, sex, race/ethnicity, PRISM-3 score, exposure to IRS, quartile of unit volume of patients with SCD, and patient origin. In the multivariable model, the analysis groups for patient origin were classified as “Admission from the Emergency Department (ED)”, “Admission from a regular floor”, “Admission from an Operating Room (OR)”, and “Other”. All analyses were performed using Stata v15.

## Supplementary Information

Supplementary Information.

## Data Availability

The data analyzed during the current study are available by appropriate request from the Virtual Pediatric Systems, LLC data registry, https://myvps.org/research/, or by appropriate request to Dr. Nicholas Ettinger, nicholas.ettinger@bcm.edu.
