# Supplementary material for: Multi-center retrospective study of children with sickle cell disease admitted to pediatric intensive care units in the United States
Source: Sci Rep. 2023 Apr 25;13:6758. doi: 10.1038/s41598-023-32651-z (PMC10130031; doi:10.1038/s41598-023-32651-z)

Multi-center Retrospective Study of Children with Sickle Cell Disease Admitted to Pediatric Intensive Care Units in the United States

SUPPLEMENTARY TABLES AND FIGURES

Table of Contents 1

Table S1. Top 10 List of Primary Diagnosis Categories 2
Table S2. Select Diagnosis Groupings 3
Table S3. Table of Primary VPS Diagnoses – Infectious Arranged by 15
Decreasing Frequency 15
Table S4. Table of Primary VPS Diagnoses – Respiratory Arranged by Decreasing Frequency 17
Table S5. Table of Primary VPS Diagnoses – Cardiac Arranged by Decreasing Frequency ... 19
Table S6. Univariate Analysis of VPS Diagnosis Categories and Resultant Odds Ratios of PICU Mortality among 5264 Admissions 21
Table S7. Univariate Analysis of SCD Patients Need for PICU Interventions and Resultant Odds Ratios of PICU Mortality – Cardiac and Respiratory 23
Table S8. Univariate Analysis of SCD Patients Need for PICU Interventions and Resultant Odds Ratios of PICU Mortality – General Surgical, Renal or Neurologic 25
Table S9. Demographic Analysis of SCD PICU Need for Invasive Respiratory Support (IRS) 27
Table S10. Demographic Analysis of SCD PICU by Stroke 29
Supplementary Figure Legends: 31

Table S1. Top 10 List of Primary Diagnosis Categories

**Primary Diagnosis Category**	**N**	**Percentage %**
Sickle Cell	2331	44.28
Respiratory	1561	29.65
Hematology	1528	29.03
Neurology	737	14
Transplant	360	6.84
Stroke	290	5.51
Infection	277	5.26
Cardiology	239	4.54
Gastroenterology/Endocrine	149	2.83
Pulmonary Hypertension	24	0.46
Pulmonary Embolism	9	0.17

Table S2. Select Diagnosis Groupings

Sickle Cell Disease
VPS Star Codes
282.61 - Sickle Cell Anemia without Crisis
282.62 - Sickle Cell Anemia with Crisis
282.6 - Sickle Cell Disease (includes both SS and SC)
517.3 - Crisis, Acute Chest Syndrome
ICD-9 Codes
282.6 - Sickle-cell disease
282.41 - Sickle-cell thalassemia without crisis
282.42 - Sickle-cell thalassemia with crisis
ICD-10 Codes:
D57 – Sickle cell disorders

Excluded:
D57.3 – sickle cell trait

Stroke:
➔For Ischemic stroke, diagnoses are labelled with I; for hemorrhagic stroke, diagnoses are labeled with H
Subdural hemorrhage/hematoma **(H)**
Nontraumatic subarachnoid hemorrhage **(H)**
Nontraumatic intracranial hemorrhage, unspecified **(H)**
Nontraumatic intracerebral hemorrhage, unspecified **(H)**
Intracranial hemorrhage NEC **(H)**
Epidural hemorrhage/hematoma **(H)**
Cerebrovascular Anomalies, Cerebral Arteriovenous Malformation **(H)**
Cerebral Aneurysm, Non-ruptured, Berry Aneurysm, Non-ruptured **(H)**
Bleeding, unknown reoperation status - Neurological/Neurosurgical System (includes Device, Graft, Prosthesis, Organ, Structure, or Shunt) **(H)**
Cerebral Artery Occlusion/Infarction **(I)**
Cerebral Artery Thrombosis/embolism **(I)**
Stroke/cerebrovascular accident **(I)**
Transient cerebral ischemia **(I)**
Acute cerebrovascular disease, ill-defined **(I)**

Pulmonary Embolism:
Pulmonary Embolism/Infarction
Septic pulmonary embolism

Pulmonary Hypertension:
Pulmonary Hypertension, Primary

Pulmonary Hypertension, Secondary or Unspecified

Respiratory:
Abscess Of Lung
Acute Chest Syndrome, Also Code with Sickle Cell
Acute Pulmonary Edema/Lung, Noncardiogenic
Acute bronchospasm
Acute laryngitis
Acute nasopharyngitis
Agenesis/Hypoplasia Of Lung, Pulmonary Aplasia, Sequestration Of Lung
Asphyxia (Not Birth or Trauma)/Hypoxemia/Hypoxic
Aspiration Pneumonitis
Asthma with acute exacerbation
Asthma with Status Asthmaticus
Asthma without Status Asthmaticus
Bronchiectasis
Bronchitis
Bronchitis/Bronchiolitis
Bronchopulmonary Dysplasia (BPD)
Choanal Atresia
Chronic disease of tonsils and/or adenoids
Chylothorax
Cleft Lip / Cleft Palate / Cleft Uvula
Congenital lung disorders
Croup/Laryngotracheobronchitis/laryngotracheitis
Cystic Fibrosis (CF)
Dental, Jaw and orthodontia disorders
Deviated nasal septum
Diseases of oral soft tissues
Diseases of tongue
Disorders of diaphragm including paralysis (excluding CDH)
Emphysema/Emphysematous Bleb
Empyema
Exercise Induced Bronchospasm
External ear disorders
Hearing disorders and other ear disorders NEC*
Impacted cerumen
Interstitial Emphysema (PIE) /Pneumomediastinum, Subcutaneous Emphysema
Laryngeal Spasm/Laryngospasm
Larynx/Sub/Supraglottic Edema
Larynx/Subglottic/Trachea/Bronchus/Airway Anomalies, includes Stenosis, Web, Atresia, Cleft, Cyst, Malacia
Mastoiditis and mastoid disorders

Mediastinal disease
Middle ear, tympanic membrane and mastoid disorders NOT mastoiditis
Obstruction, chronic airway NEC
Obstructive Sleep Apnea (OSA)
Other diseases of lower respiratory tract
Other diseases of the upper respiratory tract
Otitis Media (OM) and tube disorders
Pneumonia due to other unspecified organism
Perinatal Apnea
Perinatal Respiratory Problems, Other NEC, Congenital Pneumonia, Transitory Tachypnea Of Newborn (TTN)
Pharyngeal/Upper Airway Abscess/Cellulitis, Parapharyngeal/retropharyngeal Abscess
Pharyngitis/Tonsillitis
Pleural Effusion including Hemothorax, non-traumatic
Pneumonia due to Legionnaires Disease
Pneumonia, Bacterial
Pneumonia, Other
Pneumonia, Viral
Pneumothorax
Pulmonary Collapse/Atelectasis
Pulmonary Fibrosis, Post-inflammatory
Pulmonary Hemosiderosis
Pulmonary Insufficiency/Shock Lung, Adult Respiratory Distress Syndrome, Acute Hypoxic Respiratory Failure, Acute Lung Injury
Radiation Pneumonitis
Respiratory Complication, NEC
Respiratory Distress Syndrome, Neonatal, Hyaline Membrane Disease
Respiratory Failure/Arrest
Respiratory Syncytial Virus Pneumonia/ Pneumonitis
Respiratory System Diseases, Miscellaneous NEC
Salivary gland disease
Sinusitis
Stenosis, Larynx, Subglottic Stenosis
Trachea/Bronchus disorder NEC (Including Stenosis)
Trachea/bronchus disease NEC
Tracheitis
Tracheoesophageal Fistula/Esophageal Atresia
Tracheostomy Complication
Transfusion related acute lung injury
Upper Airway Obstruction NEC
Upper Respiratory Infection
Ventilator Associated Pneumonia
Vertigo/Vestibular System Disorders, Labyrinthitis/Menieres Disease

Vocal Cord/Larynx Paralysis

Hematology
Acquired coagulation factor deficiency (including vitamin K deficiency and liver disease)
Acquired hemolytic anemia unspecified
Anemia, Other
Aplastic and Sideroblastic Anemia
Autoimmune Hemolytic Anemia
Congenital Factor VIII Disorder, Hemophilia A
Congenital deficiency of other clotting factors including 11
Congenital factor IX disorder (hemophilia B)
Decreased white blood cell count/ Leukopenia
Defibrination Syndrome, Disseminated Intravascular Coagulation (DIC)
Disorders of glutathione metabolism including Glucose-6-p Dehydrogenase Deficiency (G6PD)
Disorders of the spleen
Ecchymoses/Petechiae
Eosinophilia
Hemoglobinopathies NEC
Hemolytic Anemia Of Enzyme Deficiency, Glucose-6-p Dehydrogenase Deficiency (G6PD) , Pyruvate Kinase Deficiency
Hemolytic hemoglobinuria from external causes
Hereditary Hemolytic Anemia excluding G6PD deficiency
Increased White Blood Cell Diseases/ Leukocytosis
Iron Deficiency Anemia and Chronic Blood Loss
Methemoglobinemia
Neutropenia and functional neutrophil disorders
Nonautoimmune Hemolytic Anemias excluding Hemolytic Uremic Syndrome
Nutritionally associated anemias, excluding iron deficiency
Other coagulation defects
Pancytopenia
Polycythemia
Primary Thrombocytopenia, Thrombocytopenic Purpura (ITP)/Evans Syndrome
Primary hypercoagulable states
Purpura/Other Hemorrhagic Conditions, Henoch-Schonlein Purpura (HSP)/ Platelet Defects
Secondary Thrombocytopenia, includes Kasabach-Merritt Thrombocytopenia
Sickle Cell Anemia With Crisis, Vaso-occlusive/painful Crisis
Sickle Cell Anemia With Crisis, Vaso-occlusive/painful Crisis, Sequestration/hemolytic/aplastic Crisis, Acute Chest Syndrome
Sickle Cell Disease (includes both SS and SC)
Splenic sequestration
Thalassemias
Von Willebrands Disease
White Blood Cell Diseases

other blood abnormalities

Neurologic:
Abnormal involuntary movement NEC
Acute Pain, includes Unspecified Pain
Acute cerebrovascular disease, ill-defined
Acute flaccid paralysis/acute flaccid myelitis
Acute transverse myelitis
Anomalies of Spinal Cord
Anoxic Brain Damage, Anoxic/Hypoxic/Ischemic Encephalopathy (HIE)
Arnold-Chiari Malformation
Ataxia, includes Spinocerebellar Ataxia and other Hereditary Ataxias
Autonomic disorders
Bacterial meningitis NOS*
Bell's Palsy/Seventh Nerve Palsy
Benign Intracranial Hypertension, Pseudotumor Cerebri
Birth Asphyxia/Intrauterine Hypoxia, Fetal Distress/Anoxia
Bleeding, unknown reoperation status - Neurological/Neurosurgical System (includes Device, Graft, Prosthesis, Organ, Structure, or Shunt)
Brain Death
Broken device, breakdown, wear, and other mechanical problems - Neurological/Neurosurgical System (includes Device, Graft, Prosthesis, Organ, Structure, or Shunt)
CNS abscess NOS
Cerebral Aneurysm, Non-ruptured, Berry Aneurysm, Non-ruptured
Cerebral Arteritis
Cerebral Artery Occlusion/Infarction, Cerebral Artery Thrombosis/embolism, Stroke/cerebrovascular Accident (CVA)
Cerebral Cysts, Porencephalic Cyst
Cerebral Degenerations Of Childhood, Cerebral Sphingolipidosis, Tay-Sachs Disease, Leighs Disease, Gangliosidosis
Cerebral Edema
Cerebral Palsy (CP)
Cerebrovascular Anomalies, Cerebral Arteriovenous Malformation (AVM)
Chorea, tremor, extrapyramidal, myoclonus, clonic spasm, and other movement disorders
Chronic Pain, includes Acute On Chronic
Coma/Stupor/Unconsciousness, Altered Mental Status (AMS)
Compression Of Brain/Herniation
Congenital Hydrocephalus, Aqueduct Of Sylvius Stenosis/obstruction, includes Dandy-Walker Malformation/Cyst and Atresia Of Foramina Of Magendie & Luschka
Congenital Nervous System Anomalies, Macroencephaly/Porencephaly
Convulsion/Seizure, Febrile Seizure/Convulsion
Cranial Nerve Disorder
Critical Illness Myopathy

Dandy-Walker Malformation/Cyst, Atresia Of Foramina Of Magendie & Luschka
Dystonia
Encephalitis/Myelitis/Encephalomyelitis
Encephalocele
Encephalopathy, Unspecified
Epidural Hemorrhage/Hematoma
Epilepsy and Epileptic Syndromes with Status Epilepticus
Epilepsy and Epileptic Syndromes without Status Epilepticus
Febrile Seizure/Convulsion
Gait Disturbance/Abnormality
Grand mal status
Guillain-Barre Syndrome
Holoprosencephaly/Anencephaly
Hydrocephalus Unspecified, includes Normal Pressure/Post-Traumatic
Hydrocephalus, Communicating Acquired
Hydrocephalus, Obstructive Acquired
Hypertensive Encephalopathy
Hypoxic Ischemic Encephalopathy (HIE)
Infarction/Compression of Spinal Cord
Infection/Inflammatory Reaction (excludes SSI, CLABSI, UTI, Pneumonia, and Other Specified Infections) - Neurological/Neurosurgical System (includes Device, Graft, Prosthesis, Organ, Structure, or Shunt)
Intracranial (includes Intraspinal) Venous Sinus Thrombosis, Sagittal Vein Thrombosis
Intracranial Abscess
Intracranial Hemorrhage NEC
Intraspinal/Paraspinal Abscess
Learning disorder
Mechanical Complication of Nervous Syst Device, Ventricular Shunt Malfunction
Meningitis, Unspecified Cause
Mental Retardation (MR), Developmental Delay, Generalized
Microcephalus/Microencephaly
Migraine
Moya moya Disease
Myasthenia Gravis
Myoneuronal Disorders, excluding Myasthenia Gravis
Myopathy/ Muscular Dystrophy, includes Duchenne, Becker, Fascioscapulohumeral, Gowers, Limb-girdle and NEC
Nervous System Anomalies, Congenital, Cerebral Cyst/Macroencephaly/Porencephaly
Nervous system anomaly NOS
Neuropathy, Inflammatory/Toxic/Other
Nontraumatic Intracerebral Hemorrhage, Parenchymal/Intraventricular Hemorrhage (IVH)
Nontraumatic Intracranial Hemorrhage NEC
Nontraumatic Subarachnoid Hemorrhage

Other Brain and CNS Conditions NEC
Other Complication - Neurological/Neurosurgical System (includes Device, Graft, Prosthesis, Organ, Structure, or Shunt)
Other cerebrovascular disease
Other demyelinating disorders of CNS
Pain not elsewhere classified
Paralytic Syndromes, Quadriplegia/paraplegia/hemiplegia
Peripheral Nerve disorders, Includes Carpal Tunnel Syndrome
Petit mal status
Pneumococcal meningitis
Post Traumatic Seizure/Convulsion
Sensation Disturbance/Paresthesia
Sleep Apnea, Unspecified
Sleep Disturbance
Specific Developmental Delay, includes Motor/Psychological Development, excludes Learning Disorder and Language or Speech Delay
Speech/language/communication disorders
Spina Bifida/Meningomyelocele
Spinal Cord Diseases, Syringomyelia/syringobulbia
Staphylococcal meningitis
Streptococcal meningitis
Subdural Hemorrhage/Hematoma
Syncope/Collapse
Thoracic Outlet Syndrome
Toxic encephalopathy
Transient cerebral ischemia
Epilepsy/seizures NEC
Organic sleep apnea (excluding obstructive)
Organic sleep disorders (excluding apneas)

Cardiac:
AV Canal (Atrioventricular septal defect)
Acute combined systolic (congestive) and diastolic (congestive) heart failure
Acute on chronic combined systolic (congestive) and diastolic (congestive) heart failure
Acute systolic (Congestive) Heart Failure
Aneurysm, Artery/Ventricle/Other
Aortic Aneurysm (includes ectasia)
Aortic Root Dilatation/Aortic Aneurysm, includes Congenital
Aortic Valve Insufficiency/Regurgitation
Aortic and Aortic Arch Anomalies/Vascular Ring/Sling, Aberrant Subclavian/double Aortic Arch
Aortic arch hypoplasia, includes absence and aplasia of aorta
Aortic stenosis, subvalvar, supravalvar and valvar

Aortic valve abnormality, incl regurgitation, atresia, combination abnormalities, other
Arrythmia, Heart Block, Acquired
Arterial Embolism/Thrombosis
Arterial dissection, not aortic, not coronaries
Arteriovenous Malformation/Aneurysm
Arteritis, Vasculitis NOS
Atherosclerosis, not cerebral or coronary vessels
Atrial Premature Contractions
Atrial Septal Defect
Atrioventricular Block, Complete
Atrioventricular Block, Incomplete
Blood vessel anomaly
Cardiac Arrest
Cardiac Dysrhythmia/Arrhythmia
Cardiac Tamponade
Cardiomyopathy
Cerebral atherosclerosis
Circulatory anomaly NOS
Coarctation of aorta
Common/Single Ventricle
Complication, Specified Procedures NEC
Complications of cerebrovascular disease or Intracranial Hemorrhage
Conduction Disorders of Heart, Interference/Atrioventricular Dissociation
Congenital Heart Block
Coronary Artery Anomaly
Coronary artery disease
Diseases of capillaries
Epstein's Anomaly
Endocardial Fibroelastosis
Endocarditis, Acute/Subacute, Bacterial Endocarditis
Heart Anomalies, Other Congenital NEC, Congenital Heart Disease NEC, Cor Tri-atriatum/Ectopia Cordis
Heart Failure, Congestive Heart Failure
Heart Transplant Status V
Heart and cardiovascular disease NEC
Hemorrhage, unspecified excludes GI, traumatic or intracranial
Hypertension NEC
Hypertension, Benign
Hypertension, Essential
Hypertension, Secondary
Hypertension, Secondary, Benign
Hypertension, Secondary, Malignant
Hypoplastic Left Heart Syndrome

Hypoplastic ventricle
Hypotension NEC
Hypovolemic Shock/Dehydration
Interruption of aortic arch
Junctional Ectopic Tachycardia
Long QT Syndrome
Major aortopulmonary collateral[s]) (without PA-VSD)
Malfunction, not working NOS, includes leakage, erosion, or exposure - Cardiac System (includes Device, Graft, Prosthesis, Organ, or Structure)
Malignant Hypertension
Mechanical Complication, Cardiac Device, Pacemaker/Heart Valve Malfunction
Mechanical Complication, Vascular Device, Dialysis Catheter Complication/malfunction, Vena Cava Filter/umbrella Complication
Mitral Valve Insufficiency/Regurgitation
Mitral Valve Stenosis
Mitral regurgitation and mitral stenosis
Mitral valve abnormality, other
Mucocutaneous Lymph Node Syndrome, Kawasaki Disease (with Cardiac Involvement)
Myocarditis
Other circulatory disease
Other disease of arteries and arterioles
Partial AV Canal, ASD primum
Partial anomalous pulmonary venous connection
Patent Ductus Arteriosus
Pericardial Effusion
Pericardial disease, other (including pneumopericardium)
Pericarditis
Phlebitis/Thrombophlebitis
Portal Vein Thrombosis
Post-cardiotomy syndrome
Postoperative bleeding
Pulmonary Artery Anomalies/Hypoplasia, Pulmonary Sling, Pulmonary AV fistula
Pulmonary Circulatory Disease
Pulmonary Embolism/Infarction
Pulmonary Hypertension, Primary
Pulmonary Hypertension, Secondary or Unspecified
Pulmonary Valve Insufficiency/Regurgitation
Pulmonary Valve Stenosis
Pulmonary atresia
Pulmonary atresia, VSD-MAPCA
Pulmonary insufficiency and pulmonary stenosis
Pulmonary valve abnormality
Pulmonary venous stenosis

Raynaud's syndrome
Rheumatic Heart Disease/Carditis
Rheumatic valve disease, insufficiency, stenosis, other
Right heart failure, unspecified
Septic arterial embolism, excludes septic pulmonary embolism
Septic pulmonary embolism
Sinoatrial/Sinus Node Dysfunction, Sick Sinus Syndrome, Wandering Atrial Pacemaker
Supraventricular Tachycardia (SVT), Paroxysmal Atrial Tachycardia
Systemic venous obstruction
Tetralogy Of Fallot
Thromboembolism/Thrombosis, includes Venous Thromboembolism - Vascular System (includes Device, Graft, Prosthesis, Organ, or Structure)
Total anomalous pulmonary venous connection
Tricuspid Valve Insufficiency/Regurgitation
Tricuspid stenosis
Tricuspid valve abnormality
Tricuspid valve regurgitation and stenosis
Truncus Arteriosus
Unspecified diastolic (congestive) heart failure
Unspecified systolic (congestive) heart failure
Vascular Comp of other Vessel
Vascular Compression from anterior spinal artery or vertebral artery
Vena Cava Thrombosis/Embolism
Venous Embolism/Thrombosis, Other
Ventricular septal defect + Aortic arch hypoplasia
Ventricular Fibrillation/Flutter
Ventricular Premature Contractions
Ventricular Septal Defect
Ventricular Tachycardia
Wolff-Parkinson-White Syndrome

Transplant:
Bone Marrow Transplant (BMT) Status V
Complications of bone marrow transplant including VOD, GVHD
Complication of Transplanted Organ/Tissue, Graft Rejection/Engraftment Failure
Complication of bone marrow includes rejection, infection, failure, excludes VOD/SOS
Complication of heart transplant including rejection, infection, or graft failure
Complication of kidney transplant including rejection, infection, or graft failure
Complication of liver transplant including rejection, infection, or graft failure
Complication of other transplant including rejection, infection, or graft failure
Graft-Versus-Host Disease (GVHD), excludes VOD/SOS
Kidney Transplant Status
Liver Transplant Status

Stem Cell Transplant Status

Infectious:
Adenovirus Infection
Anaerobic septicemia
Aspergillus Infection
Bacteremia, Unspecified
Bacterial Infection, Other
Bacteroides Fragilis Infection
CNS infection, Viral Not Arthropod-Borne, excludes enteroviral
Candida Infection, NEC
Candidal esophagitis
Chlamydiae Infection
Clostridium Difficile Infection, Pseudomembranous Colitis
Congenital cytomegalovirus infection
Coxsackie Virus Infection
Cysticercosis
Cytomegalovirus Infection
Disseminated candidiasis
E coli septicemia
Enterococcus group d infection
Enteroviral Infection (Includes Coxsackie, Echovirus, Parechovirus) excludes Polio and Enteroviral Meningitis
Epstein-Barr Virus Infection (EBV), Infectious Mononucleosis
Escherichia Coli Infection
Gangrene
Gastroenteritis/Colitis, Infectious, Diarrhea, Infectious
Gram-negative septicemia NOS
Hemophilus influenzae septicemia
H1N1 influenza virus (Swine Flu)
Hemophilus Influenzae Infection and other Hemophilus species
Hepatitis in viral dis
Herpes Virus includes Herpes Simplex, Human Herpes
Herpes zoster
Indwelling Catheter/Broviac Infection
Infection Associated w/ Central Venous Catheter
Influenza Virus Infection
Klebsiella Infection, Friedlanders Bacillus Infection
Lymphadenitis
Malaria Infection
Methicillin Resistant Staphylococcus Aureus - unspecified infection
Methicillin Resistant Staphylococcus Aureus Septicemia
Mucocutaneous Lymph Node Syndrome, Kawasaki Disease

Mucocutaneous Lymph Node Syndrome, Kawasaki Disease (without Cardiac Involvement)
Mycoplasma Infection
Mycoses, Other NEC, Fungal Infection, Other
Other Mycobacteria Infection, not Tuberculosis
Other gram negative bacteria, including Serratia
Other viral infections
Parasitic Infections, Other
Parvovirus Infection, includes B19
Pneumococcal Infection
Pneumococcal septicemia
Pneumocystis Carinii Pneumonia
Pneumonia Due To SARS
Pneumonia due to Methicillin Resistant Staphylococcus Aureus
Proteus Infection
Pseudomonas Infection
Pseudomonas septicemia
Pyogenic/Septic Arthritis
SARS and other diseases associated with SARS Coronavirus
Salmonella Infection
Sarcoidosis
Sepsis or Severe Sepsis (no Septic shock)
Septic Shock
Septicemia NEC
Serratia septicemia
Staphylococcal septicemia NOS
Staphylococcal Infection (Excludes Sepsis)
Methicillin Sensitive Staphylococcus Aureus Infection
Methicillin Sensitive Staphylococcus Aureus Septicemia
Streptococcal Infection, NEC
Streptococcal septicemia
Streptococcus group A infection
Streptococcus group B infection
Toxic Shock Syndrome
Toxoplasmosis/Toxoplasma Gondii Infection
Unspecified infectious disease
Viral Exanthemata, Other, Erythema Infectiosum/Exanthema Subitum
Viral Hepatitis Infection, Includes (HAV)(HBV)(HCV) and others
Viral Infection, Arthropod-Borne, Viral Encephalitis/hemorrhagic Fever, Arbovirus Infection
Viral Infection, Other NEC
Viral Warts
Viremia, Unspecified
Sepsis with or without shock

[*] - NEC - Not elsewhere classifiable, NOS – Not otherwise specified

Table S3. Table of Primary VPS Diagnoses – Infectious Arranged by Decreasing Frequency

Primary VPS Diagnosis - Infectious	Frequency (N)	Percentage (%)
**Sepsis with or without shock**	**102**	**36.82**
**Other viral infections**	**40**	**14.44**
**Septic shock**	**29**	**10.47**
**Influenza Virus Infection**	**21**	**7.58**
**Bacteremia, Unspecified**	**8**	**2.89**
**Septicemia NEC**	**8**	**2.89**
**Infection Associated with Central Venous Catheter**	**6**	**2.17**
**Bone Infections/Osteomyelitis/Periostitis**	**5**	**1.81**
**Gram-negative septicemia NOS**	**5**	**1.81**
**Salmonella Infection**	**5**	**1.81**
Escherichia coli septicemia	4	1.44
Sepsis or Severe Sepsis (no septic shock)	4	1.44
Adenovirus Infection	3	1.08
H1N1 influenza virus (Swine Flu)	3	1.08
Parvovirus Infection, includes B19	3	1.08
Pneumococcal septicemia	3	1.08
Cytomegalovirus Infection	2	0.72
Gastroenteritis/Colitis, Infectious, Diarrhea, Infectious	2	0.72
Hemophilus influenzae septicemia	2	0.72
Indwelling Catheter/Broviac Infection	2	0.72
Malaria Infection	2	0.72
Methicillin Resistant Staphylococcus Aureus - unspecified infection	2	0.72
Mycoses, Other NEC, Fungal Infection, Other	2	0.72
Streptococcus Group A infection	2	0.72
Aspergillus Infection	1	0.36
Candida Infection, NEC	1	0.36
Clostridium Difficile Infection, Pseudomembranous Colitis	1	0.36
Cysticercosis	1	0.36
Mucocutaneous Lymph Node Syndrome, Kawasaki Disease	1	0.36
Mucocutaneous Lymph Node Syndrome, Kawasaki Disease (without Cardiac Involvement)	1	0.36
Other Mycobacteria Infection, not Tuberculosis	1	0.36
Pneumocystis Carinii Pneumonia	1	0.36
Methicillin Sensitive Staphylococcus Aureus Septicemia	1	0.36
Streptococcal septicemia	1	0.36

Viral Infection, Arthropod-Borne, Viral Encephalitis/hemorrhagic Fever, Arbovirus Infection	1	0.36
Viral Infection, Other NEC	1	0.36
**Total**	**277**	**100**

Table S4. Table of Primary VPS Diagnoses – Respiratory Arranged by Decreasing Frequency

Primary VPS Diagnosis - Respiratory	Frequency (N)	Percentage (%)
**Acute Chest Syndrome, Also Code with Sickle Cell**	**930**	**59.58**
**Pneumonia, Other**	**99**	**6.34**
**Asthma With Status Asthmaticus**	**96**	**6.15**
**Pulmonary Insufficiency/Shock Lung, Adult Respiratory Distress Syndrome, Acute Hypoxic Respiratory Failure, Acute Lung Injury**	**79**	**5.06**
**Obstructive Sleep Apnea**	**75**	**4.8**
**Bronchitis/Bronchiolitis**	**51**	**3.27**
**Asthma with acute exacerbation**	**23**	**1.47**
**Chronic disease of tonsils and/or adenoids**	**21**	**1.35**
**Pneumonia, Bacterial**	**17**	**1.09**
**Respiratory Syncytial Virus (RSV) Pneumonia/Pneumonitis**	**14**	**0.9**
Asphyxia (Not Birth or Trauma)/Hypoxemia/Hypoxic	12	0.77
Pneumonia, Viral	12	0.77
Respiratory Failure/Arrest	11	0.7
Tracheitis	10	0.64
Stenosis, Larynx, Subglottic Stenosis	9	0.58
Croup/Laryngotracheobronchitis/laryngotracheitis	8	0.51
Other diseases of lower respiratory tract	8	0.51
Larynx/Subglottic/Trachea/Bronchus/Airway Anomalies, includes Stenosis, Web, Atresia, Cleft, Cyst, Malacia	6	0.38
Aspiration Pneumonitis	5	0.32
Bronchiolitis due to RSV	5	0.32
Bronchiolitis, excludes RSV	5	0.32
Bronchitis	5	0.32
Pleural Effusion including hemothorax, non-traumatic	5	0.32
Upper Respiratory Infection	4	0.26
Dental, Jaw and orthodontia disorders	3	0.19
Hearing disorders and other ear disorders NEC	3	0.19
Pneumothorax	3	0.19
Trachea/Bronchus disorder NEC (including stenosis)	3	0.19
Transfusion related acute lung injury	3	0.19
Upper Airway Obstruction NEC	3	0.19
Abscess of lung	2	0.13
Asthma without status asthmaticus	2	0.13
Other diseases of the upper respiratory tract	2	0.13

Pharyngeal/Upper Airway Abscess/Cellulitis, Parapharyngeal/retropharyngeal Abscess	2	0.13
Pharyngitis/Tonsillitis	2	0.13
Respiratory Complication, NEC	2	0.13
Respiratory System Diseases, Miscellaneous NEC	2	0.13
Acute Pulmonary Edema/Lung, Noncardiogenic	1	0.06
Acute Pulmonary Insufficiency/Respiratory Failure - airway and respiratory	1	0.06
Bleeding, unknown reoperation status - airway and respiratory	1	0.06
Bronchiectasis	1	0.06
Chylothorax	1	0.06
Empyema	1	0.06
Laryngeal Spasm/Laryngospasm	1	0.06
Larynx/Sub/Supraglottic Edema	1	0.06
Mediastinal disease	1	0.06
Obstruction, chronic airway NEC	1	0.06
Other Complication - Airway and Respiratory	1	0.06
Otitis Media and tube disorders	1	0.06
Perinatal Apnea	1	0.06
Pneumonia due to Legionnaires Disease	1	0.06
Pulmonary Collapse/Atelectasis	1	0.06
Pulmonary Hemosiderosis	1	0.06
Respiratory conditions due to smoke inhalation	1	0.06
Sinusitis	1	0.06
Vertigo/Vestibular System Disorders, Labyrinthitis/Meniere’s Disease	1	0.06
**Total**	**1,561**	**100**

Table S5. Table of Primary VPS Diagnoses – Cardiac Arranged by Decreasing Frequency

Primary VPS Diagnosis - Cardiac	Frequency (N)	Percentage (%)
**Hypotension NEC***	**18**	**7.53**
**Pulmonary Hypertension, Secondary or Unspecified**	**16**	**6.69**
**Hypertension NEC**	**15**	**6.28**
**Cardiac Arrest**	**12**	**5.02**
**Pericardial Effusion**	**12**	**5.02**
**Cardiomyopathy**	**11**	**4.6**
**Venous Embolism/Thrombosis, Other**	**11**	**4.6**
**Hypovolemic Shock/Dehydration**	**10**	**4.18**
**Ventricular Septal Defect**	**10**	**4.18**
**Pulmonary Embolism/Infarction**	**9**	**3.77**
Heart Failure, Congestive Heart Failure	8	3.35
Pulmonary Hypertension, Primary	8	3.35
Supraventricular Tachycardia, Paroxysmal Atrial Tachycardia	8	3.35
Conduction Disorders of Heart, Interference/Atrioventricular Dissociation	7	2.93
Malignant Hypertension	5	2.09
Vena Cava Thrombosis/Embolism	5	2.09
Arterial Embolism/Thrombosis	4	1.67
Complication, Specified Procedures NEC	4	1.67
Heart and cardiovascular disease NEC	4	1.67
Hypertension, Essential	4	1.67
Other circulatory disease	4	1.67
Common/Single Ventricle	3	1.26
Endocarditis, Acute/Subacute, Bacterial Endocarditis	3	1.26
Hypertension, Secondary	3	1.26
Patent Ductus Arteriosus	3	1.26
Pericardial disease, other (including pneumopericardium)	3	1.26
Tetralogy Of Fallot	3	1.26
Aortic stenosis, subvalvar, supravalvar and valvar	2	0.84
Atrial Septal Defect	2	0.84
Complications of cerebrovascular disease or Intracranial Hemorrhage	2	0.84
Coronary Artery Anomaly	2	0.84
Major aortopulmonary collateral[s]) (without PA-VSD)	2	0.84
Mechanical Complication, Cardiac Device, Pacemaker/Heart Valve Malfunction	2	0.84

Mechanical Complication, Vascular Device, Dialysis Catheter Complication/malfunction, Vena Cava Filter/umbrella Complication	2	0.84
Total anomalous pulmonary venous connection	2	0.84
Aneurysm, Artery/Ventricle/Other	1	0.42
Aortic and Aortic Arch Anomalies/Vascular Ring/Sling, Aberrant Subclavian/double Aortic Arch	1	0.42
Aortic arch hypoplasia, includes absence and aplasia of aorta	1	0.42
Arrythmia, Heart Block, Acquired	1	0.42
Arterial dissection, not aortic, not coronaries	1	0.42
Arteritis, Vasculitis NOS	1	0.42
Cerebral atherosclerosis	1	0.42
Coarctation of aorta	1	0.42
Mitral Valve Insufficiency/Regurgitation	1	0.42
Mitral valve abnormality, other	1	0.42
Partial AVC (AVSD), ASD primum	1	0.42
Partial anomalous pulmonary venous connection	1	0.42
Pericarditis	1	0.42
Postoperative bleeding	1	0.42
Pulmonary Circulatory Disease	1	0.42
Pulmonary atresia, VSD-MAPCA	1	0.42
Pulmonary valve abnormality	1	0.42
Rheumatic valve disease, insufficiency, stenosis, other	1	0.42
Vascular Complication of other Vessel	1	0.42
Vascular Compression from anterior spinal artery or vertebral artery	1	0.42
**Total**	**239**	**100**

Table S6. Univariate Analysis of VPS Diagnosis Categories and Resultant Odds Ratios of PICU Mortality among 5264 Admissions

VPS Diagnoses†	Number of Admissions with Data	Total Number of Admissions with Positive Exposure	Odds Ratio*	95% Confidence Interval		p-value
Sickle Cell–Primary	5264	2331	**0.23**	0.13	0.4	**<0.001**
Cardiology						
Primary	5264	239	**4.28**	2.35	7.79	**<0.001**
Any	5264	1261	**11.55**	6.51	20.5	**<0.001**
Infection						
Primary	5264	277	**4.38**	2.5	7.66	**<0.001**
Any	5264	1798	**4.29**	2.73	6.73	**<0.001**
Respiratory						
Primary	5264	1561	**0.43**	0.24	0.74	**0.003**
Any	5264	3079	**3.27**	1.88	5.68	**<0.001**
Neurology						
Primary	5264	737	0.83	0.49	1.61	0.700
Any	5264	1791	**3.51**	2.27	5.42	**<0.001**
Transplant						
Primary	5264	360	0	0	0	0.976
Any	5264	571	**5.41**	2.44	12.01	**<0.001**
Stroke (all)						
Primary	5264	667	0.82	0.33	2.08	0.682
Any	5264	692	**2.44**	1.49	3.97	**<0.001**
Hemorrhagic Stroke						
Primary	5264	49	**4.51**	1.50	13.53	**0.007**
Any	5264	134	**9.55**	4.53	20.13	**<0.001**
Ischemic Stroke						
Primary	5264	241	0.19	0.03	1.36	0.098
Any	5264	564	1.35	0.75	2.43	0.318
Pulmonary Hypertension						
Primary	5264	24	**6.80**	1.71	27.05	**0.007**
Any	5264	163	**3.85**	1.56	9.49	**0.003**
Pulmonary Embolism						
Primary	5264	9	5.20	0.58	46.88	0.142
Any	5264	31	**5.06**	1.32	19.38	**0.018**

[*] - Odds ratios for mortality calculated with a mixed effect logistic regression model accounting for correlations within each patient (i.e., multiple admissions per patient) and within each institution (i.e., “center effect”).

[†] - VPS diagnosis groupings listed in Supplementary Tables 1 and 2

Table S7. Univariate Analysis of SCD Patients Need for PICU Interventions and Resultant Odds Ratios of PICU Mortality – Cardiac and Respiratory

Variable	Number of Admissions with Recorded Data	Number of Admissions with Positive Exposure	Odds Ratio[*]	95% Confidence Interval		p-value
**PRISM 3 Score**	5264	5264	**1.21**	1.18	1.23	**<0.001**
**Hospital LOS (d)** [†]	5264	5264	**1.01**	1.003	1.01	**0.001**
**PICU LOS (d)** [†]	5264	5264	**1.03**	1.02	1.04	**<0.001**
**MV Duration (d)** [†]	5264	5264	**1.03**	1.01	1.04	**<0.001**
**Cardiac**						
Echocardiogram	1009	186	**7.24**	2.66	19.70	**<0.001**
Pericardiocentesis	1566	11	0	0	0	0.993
ECMO[‡]	5249	22	**30.15**	10.09	90.04	**<0.001**
CPR/Cardioversion/ Defibrillation[‡]	3012	46	**240.71**	44.23	1310.04	**<0.001**
**Respiratory**						
NIPPV[‡]	2113	1561	1.06	0.51	2.20	0.885
HFNC[‡]	4090	936	0.86	0.49	1.52	0.613
IRS[‡]	4093	872	**45.96**	21.61	97.74	**<0.001**
Endotracheal Intubation	5264	795	**42.96**	23.26	79.37	**<0.001**
Tracheostomy	3565	89	**3.49**	1.17	10.41	**0.025**
HFOV[‡]	5264	58	**24.71**	10.87	56.16	**<0.001**
Bronchoscopy	3543	74	**3.89**	1.39	10.95	**0.010**
VATS[‡]	2154	3	0	0	0	0.986
Chest Tube	2773	87	**4.66**	1.90	11.46	**0.001**
Angiography	975	36	2.70	0.60	12.12	0.194
V/Q scan	936	1	0	0	0	0.992
iNO[‡]	2169	83	**7.89**	3.21	19.38	**<0.001**
Heliox	2153	14	0	0	0	0.986
**Imaging/Lines**						
CT scan[‡]	1019	113	**7.23**	3.05	17.16	**<0.001**
MRI scan[‡]	1042	167	1.34	0.45	4.02	0.600
CVC/Port/PICC[‡]	5264	1539	**12.80**	7.33	22.37	**<0.001**
Arterial Line	5264	837	**14.07**	8.88	22.27	**<0.001**
Intraosseous Catheter	2489	14	**29.33**	9.23	93.21	**<0.001**

[*] - Odds ratios for mortality calculated with a mixed effect logistic regression model accounting for correlations within each patient (multiple admissions per patient) and accounting for correlations for within each institution (i.e., “center effect”).

† – Odds ratio for mortality for a 1-day increase in PICU LOS, hospital LOS, or length of mechanical ventilation

‡ - Abbreviations: ECMO – extracorporeal membrane oxygenation; CPR – cardiopulmonary resuscitation; NIPPV – non-invasive positive pressure ventilation; HFOV – high frequency oscillator ventilation; VATS – video-assisted thoracoscopic surgery; HFNC – high flow nasal cannula; IRS – invasive respiratory support; iNO – inhaled nitric oxide; V/Q scan – ventilation-perfusion scan; CT – computed tomography; MRI – magnetic resonance imaging; CVC – central venous catheter; PICC – peripherally inserted central catheter

Table S8. Univariate Analysis of SCD Patients Need for PICU Interventions and Resultant Odds Ratios of PICU Mortality – General Surgical, Renal or Neurologic

Variable	Number of Admissions with Recorded Data	Number of Admissions with Positive Exposure	Odds Ratio*	95% Confidence Interval		p-value
**General Surgical**						
Gastrostomy Tube	1247	42	2.96	0.33	26.57	0.333
Orogastric Tube	1233	4	0.00	0.00	0.00	0.995
NG tube†	1259	107	**5.48**	1.59	18.92	**0.007**
ND/NJ tube†	1239	23	**18.05**	2.08	156.59	**0.009**
Bone Marrow Biopsy	1400	7	0.00	0.00	0.00	0.988
Paracentesis	1580	2	0	0	0	0.989
Appendectomy	2109	7	6.59	0.74	58.50	0.090
Splenectomy	2114	28	3.19	0.72	14.13	0.127
Laparotomy	2119	34	**3.78**	1.09	13.09	**0.036**
**Renal**						
Renal Replacement Therapy‡	5264	68	**23.73**	9.60	58.67	**<0.001**
Exchange Transfusion/ Apheresis-Plasmapheresis	3052	548	0.97	0.47	1.99	0.931
IHD†	3127	25	0.00	0.00	0.00	0.982
Hemodialysis/ Plasmapheresis Catheter	5264	1072	**2.31**	1.42	3.77	**0.001**
CRRT†	3115	25	**40.96**	11.42	146.95	**<0.001**
Peritoneal Dialysis	3117	7	0	0	0	0.986
Peritoneal Dialysis Catheter	2157	5	0	0	0	0.992
**Neurologic**						
Lumbar Puncture	1644	48	0.81	0.11	6.12	0.842
Shunt Tap	1975	1	0.00	0.00	0.00	0.995
Craniotomy	2427	108	0.72	0.16	3.25	0.675
Cerebrovascular Coiling	1674	4	0	0	0	0.988
Aneurysm Repair	2389	26	0	0	0	0.987
Cerebral Spinal Fluid diversion	2386	11	0.00	0.00	0.00	0.991

Intracranial Pressure monitoring	2688	42	**7.85**	2.15	28.68	**0.002**
EEG Monitoring†	1937	115	**3.40**	1.40	8.30	**0.007**
Pentobarbital coma	1494	2	**42.34**	1.30	1380.78	**0.035**
Hypothermic therapy	1494	3	**98.63**	2.68	3629.71	**0.013**

* - Odds ratios for mortality calculated with a mixed effect logistic regression model accounting for correlations within each patient (multiple admissions per patient) and accounting for correlations for within each institution (i.e., "center effect").

† - Abbreviations: NG – nasogastric, ND – nasoduodenal, NJ – nasojejunal, CRRT – continuous renal replacement therapy, IHD – intermittent hemodialysis, EEG – electroencephalogram

‡ - Renal Replacement Therapy - composite of CRRT, intermediate hemodialysis, or PD

Table S9. Demographic Analysis of SCD PICU Need for Invasive Respiratory Support (IRS)

Variable	No IRS (n=3221)		IRS (n=872)		
	**Median**	**(IQR)**	**Median**	**(IQR)**	**p-value***
**Weight (kg)**	29.2	(18.7,48.1)	25.1	(15.8,41.3)	**<0.001**
**Height (cm)** †	133	(112.0,153.0)	127	(104.4,152.5)	**0.011**
**Median Number of Sickle Cell Admissions per PICU**	90	(42.0,178.0)	96.5	(48.0,168.0)	0.579
	**N**	**%**	**N**	**%**	**p-value**
**Age**					**<0.001**
Infant (30d - <2 yr)	262	8.1	131	15.0	
Child (2 - <6 yr)	633	19.7	185	21.2	
Child (6 - <12 yr)	1160	36.0	311	35.7	
Adolescent (12-18 yr)	1166	36.2	245	28.1	
**Gender**					0.208
Female	1567	48.6	403	46.2	
Male	1654	51.4	469	53.8	
**Race**					**<0.001**
Black	2507	77.8	665	76.3	
Hispanic	208	6.5	18	2.1	
Other/Mixed	194	6.0	38	4.4	
Unspecified/Missing	312	9.7	151	17.3	
**Patient Origin Prior to PICU Admission**‡					**<0.001**
Regular Floor	1033	32.1	282	32.3	
ED	922	28.6	242	27.8	
Transfer from OSH	377	11.7	151	17.3	
Operating Room	360	11.2	116	13.3	
Home/Other/Skilled Nursing	396	12.3	15	1.7	
Step Down Unit	90	2.8	44	5.0	
Procedure Suite	39	1.2	13	1.5	
Another ICU/NICU	4	0.1	9	1.0	
**Geographic Patient Origin (US Census Region)**					0.149
South	1612	50.0	485	55.6	
Midwest	789	24.5	207	23.7	
West	411	12.8	89	10.2	
Northeast	333	10.3	71	8.1	

International	76	2.4	20	2.3	
**Number of Sickle Cell Admissions per PICU - By Quartiles**					**<0.001**
Q1- 1-43	893	27.7	203	23.3	
Q2 - 44-94	888	27.6	233	26.7	
Q3 - 95-178	670	20.8	311	35.7	
Q4 - 179-474	770	23.9	125	14.3	
**PICU Outcome**					**<0.001**
Died	8	0.2	86	9.9	
Survived	3213	99.8	786	90.1	

* - Data analyzed with Two-sample Wilcoxon rank-sum (Mann-Whitney) [medians] or exact testing or Chi-Square (categorical data)

† – Height data: No IRS, n=2090, IRS, n=417 due to missing data

‡ - Abbreviations in Table S4.

Table S10. Demographic Analysis of SCD PICU by Stroke

Variable	No Stroke (n=4607)		Stroke (n=657)		
	**Median**	**(IQR)**	**Median**	**(IQR)**	**p-value***
**Weight (kg)**	27.9	(17.5, 46.9)	30	(19.9,46.8)	**0.002**
**Height (cm)** †	131.1	(109.0,153.0)	134	(114.5,153.0)	**0.050**
**Median Number of Sickle Cell Admissions per PICU**	90	(43.0,178.0)	101	(47.0,168.0)	0.235
	**N**	**%**	**N**	**%**	**p-value**
**Age**					**<0.001**
Infant (30d - <2 yr)	462	10.0	19	2.9	
Child (2 - <6 yr)	1002	21.7	123	18.7	
Child (6 - <12 yr)	1578	34.3	279	42.5	
Adolescent (12-18 yr)	1565	34.0	236	35.9	
**Gender**					0.616
Female	2168	47.1	302	46.0	
Male	2439	52.9	355	54.0	
**Race**					0.099
Black	3515	76.3	495	75.3	
Hispanic	216	4.7	33	5.0	
Other/Mixed	266	5.8	26	4.0	
Unspecified/Missing	610	13.2	103	15.7	
**Patient Origin Prior to PICU Admission**‡					**<0.001**
Regular Floor	1543	33.5	151	23.0	
ED	1329	28.8	226	34.4	
Transfer from OSH	591	12.8	105	16.0	
Operating Room	508	11.0	108	16.4	
Home/Other/Skilled Nursing	416	9.0	19	2.9	
Step Down Unit	164	3.6	21	3.2	
Procedure Suite	43	0.9	25	3.8	
Another ICU/NICU	13	0.3	2	0.3	
**Geographic Patient Origin (US Census Region)**					0.380
South	2511	54.5	378	57.5	
Midwest	1050	22.8	134	20.4	
West	562	12.2	75	11.4	
Northeast	383	8.3	51	7.8	
International	101	2.2	19	2.9	

**Number of Sickle Cell Admissions per PICU - By Quartiles**					**<0.01**
Q1- 1-43	1227	26.6	156	23.7	
Q2 - 44-94	1180	25.6	148	22.5	
Q3 - 95-178	1189	25.8	223	33.9	
Q4 - 179-474	1011	21.9	130	19.8	
**PICU Outcome**					**<0.001**
Died	70	1.5	25	3.8	
Survived	4537	98.5	632	96.2	

[*] - Data analyzed with Two-sample Wilcoxon rank-sum (Mann-Whitney) [medians] or exact testing or Chi-Square (categorical data)

[†] – Height data: No IRS, n=2456, IRS, n=342 due to missing data

[‡] - Abbreviations in Table S4.

Supplementary Figure Legends:

Supplementary Figure 1: Plot of number of patients with SCD per year in the VPS database 2012-2019

(1a) – Total number of PICU admissions with SCD plotted over time (squares) vs. the total number of VPS admissions plotted over time (circles). The percentage of total admissions represented by PICU admissions with SCD is plotted as a dotted line at the bottom (triangles). Left vertical axis represents the number of admissions. Right vertical axis represents percent of total. X-axis represents time by years. Note the left Y-axis is a discontinuous axis.

(1b) – Total number of unique patients with SCD plotted over time (squares) vs. the total number of unique VPS patients plotted over time (circles). The percentage of total unique patients represented by patients with SCD is plotted as a dotted line at the bottom (triangles). Left vertical axis represents the number of patients. Right vertical axis represents percent of total. X-axis represents time by years. Note the left Y-axis is a discontinuous axis.

Supplementary Figure 2: Histogram of number of patients with SCD per center contributed to VPS 2012-2019 displayed by center (N=138)

Vertical axis represents number of PICU patients with SCD per center. Horizontal axis represents all centers contributing data to the VPS database displayed from smallest to largest number of admissions per center. Red vertical lines represent quartiles of center volume.

Supplementary Figure 1. Plot of number of patients with SCD per year in the VPS database 2012-2019

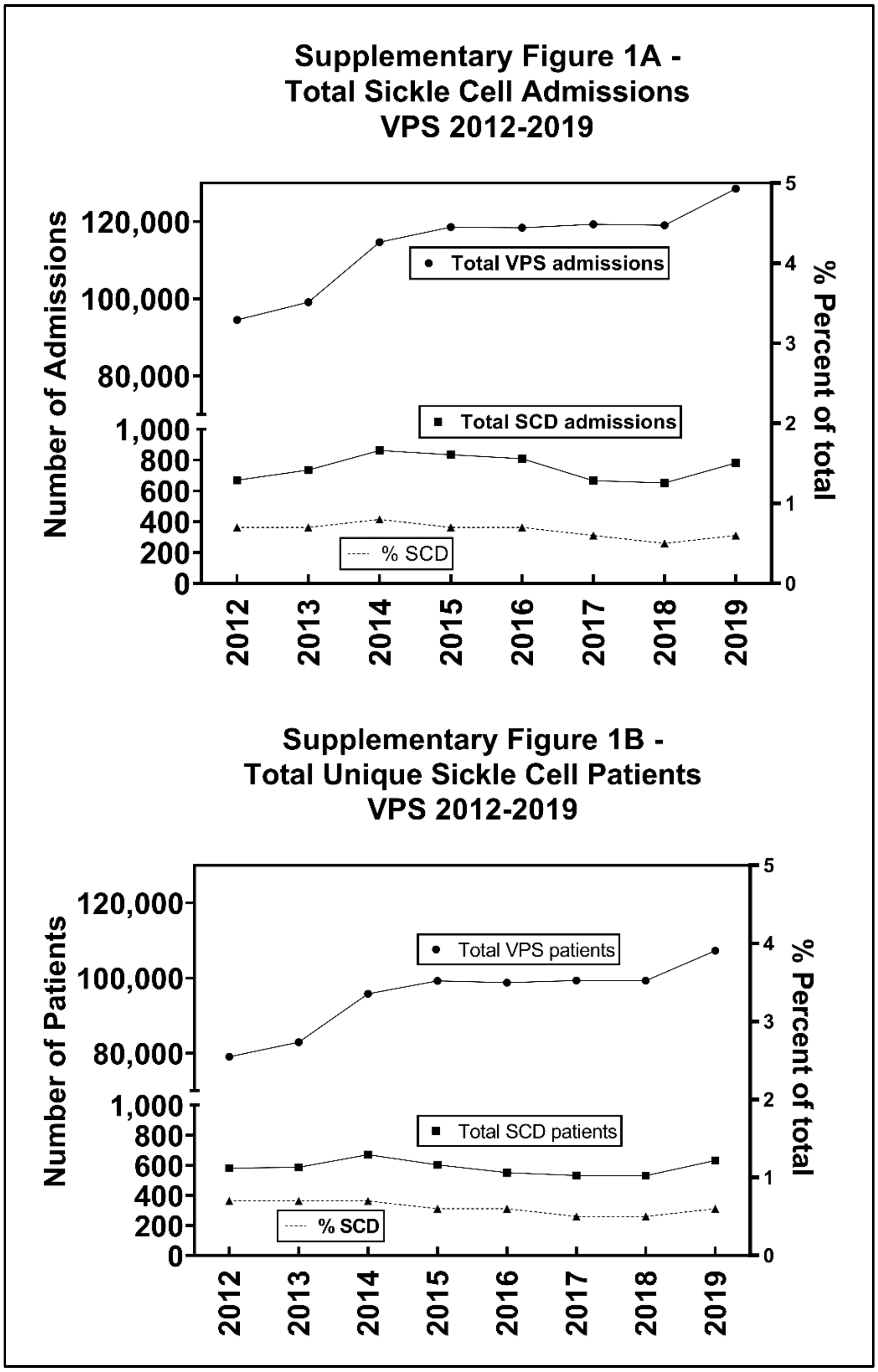

Supplementary Figure 2. Histogram of number of patients with SCD per center contributed to VPS 2012-2019 displayed by center (N=138)

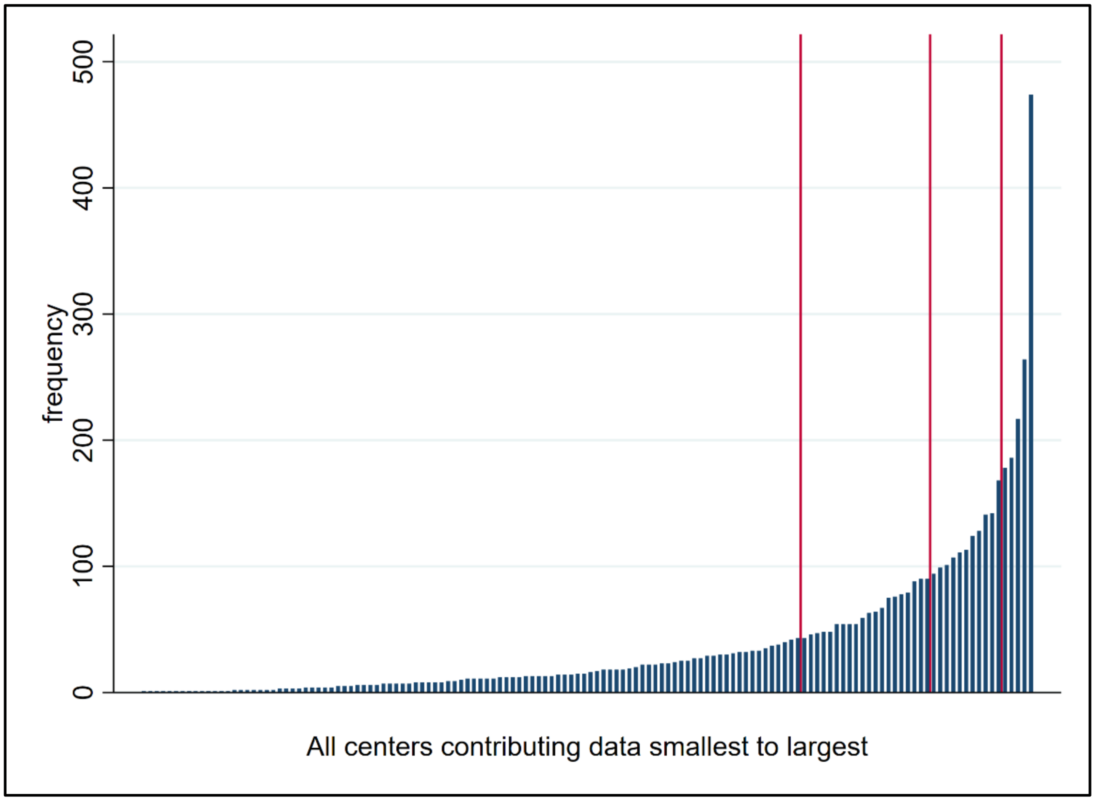

Supplement: Supplementary file 1 — Supplementary Information. [file 41598_2023_32651_MOESM1_ESM.pdf]
